# Predicting Deep Venous Thrombosis Using Artificial Intelligence: A Clinical Data Approach

**DOI:** 10.3390/bioengineering11111067

**Published:** 2024-10-25

**Authors:** Aurelian-Dumitrache Anghele, Virginia Marina, Liliana Dragomir, Cosmina Alina Moscu, Mihaela Anghele, Catalin Anghel

**Affiliations:** 1 Department of General Surgery, Faculty of Medicine and Pharmacy, “Dunărea de Jos” University, 47 Str. Domnească, 800201 Galati, Romania; anghele_aurelian@yahoo.com; 2 Medical Department of Occupational Health, Faculty of Medicine and Pharmacy, “Dunărea de Jos” University, 47 Str. Domnească, 800201 Galati, Romania; 3 Clinical-Medical Department, Faculty of Medicine and Pharmacy, “Dunărea de Jos” University, 47 Str. Domnească, 800201 Galati, Romania; lilianadragomir2017@gmail.com (L.D.); mihaela.anghele@ugal.ro (M.A.); 4 Emergency Department, “Dunărea de Jos” University, 800201 Galati, Romania; cosmina_caluian@yahoo.com; 5 Department of Computer Science and Information Technology, “Dunărea de Jos” University, 2 Științei St., 800146 Galati, Romania; catalin.anghel@ugal.ro

**Keywords:** deep venous thrombosis, machine learning, machine learning models in healthcare, artificial intelligence in medical diagnosis

## Abstract

Deep venous thrombosis is a critical medical condition that occurs when a blood clot forms in a deep vein, usually in the legs, and can lead to life-threatening complications such as pulmonary embolism if not detected early. Hospitalized patients, especially those with immobility or post-surgical recovery, are at higher risk of developing deep venous thrombosis, making early prediction and intervention vital for preventing severe outcomes. In this study, we evaluated the following eight machine learning models to predict deep venous thrombosis risk: logistic regression, random forest, XGBoost, artificial neural networks, k-nearest neighbors, gradient boosting, CatBoost, and LightGBM. These models were rigorously tested using key metrics, including accuracy, precision, recall, F1-score, specificity, and receiver operating characteristic curve, to determine their effectiveness in clinical prediction. Logistic regression emerged as the top-performing model, delivering high accuracy and an outstanding receiver operating characteristic curve score, which reflects its strong ability to distinguish between patients with and without deep venous thrombosis. Most importantly, the model’s high recall underscores its ability to identify nearly all true deep venous thrombosis cases, significantly reducing the risk of false negatives—a critical concern in clinical settings, where delayed or missed diagnoses can result in life-threatening complications. Although models such as random forest and eXtreme Gradient Boosting also demonstrated competitive performances, logistic regression proved the most reliable across all metrics. These results suggest that machine learning models, particularly logistic regression, have great potential for early deep venous thrombosis detection, enabling timely clinical interventions and improved patient outcomes.

## 1. Introduction

Deep venous thrombosis (DVT) is a significant and potentially life-threatening condition, characterized by the formation of blood clots in deep veins, most commonly in the lower extremities. DVT can lead to severe complications, such as pulmonary embolism (PE), when a part of the clot dislodges and travels to the lungs, posing a high risk of morbidity and mortality [1]. The clinical implications of DVT are profound, including prolonged hospital stays, increased healthcare costs, and the potential for long-term morbidity due to post thrombotic syndrome (PTS) [2,3]. Despite advances in preventive strategies, such as anticoagulant therapy and mechanical prophylaxis, DVT remains a persistent challenge in clinical practice, particularly in hospitalized patients who are immobile or undergoing surgery [4].

The early identification of DVT is crucial for initiating timely treatment, which significantly improves patient outcomes. However, diagnosing DVT is often complex due to the nonspecific nature of its symptoms, such as leg pain and swelling, which can overlap with other conditions [5]. Conventional diagnostic methods, including clinical evaluation, Doppler ultrasound imaging, and blood tests, such as D-dimer, provide valuable information but are not always sufficient for early detection, especially in high-risk populations [6,7]. The sensitivity and specificity of these tests can vary, leading to potential misdiagnosis or delayed treatment, which can exacerbate patient outcomes and increase the risk of PE [8].

In recent years, the application of machine learning (ML) in healthcare has emerged as a promising approach to enhance diagnostic accuracy and predict complex medical conditions. ML models have the ability to process large, multidimensional datasets, identifying patterns and correlations that may not be apparent using traditional statistical methods. These capabilities are particularly relevant in the context of DVT, where the integration of clinical, demographic, and laboratory data could improve predictive models for early detection [9]. Several studies have demonstrated the potential of ML in predicting complications such as sepsis, postoperative infections, and cardiovascular events, utilizing various algorithms such as logistic regression (LR), random forest (RF), and support vector machines (SVMs) [10,11]. However, the application of ML in predicting DVT remains relatively underexplored, especially in hospitalized patient populations.

This study aims to address this gap by systematically evaluating the performance of various supervised ML models in predicting DVT in hospitalized patients. Using a dataset of clinical data, including demographic information, medical history, and laboratory results, we compare the performance of several models, such as LR, RF, extreme gradient boosting (XGB), artificial neural networks (ANNs), k-nearest neighbors (kNN), and Gradient Boosting (GB). Each model is evaluated using key performance metrics, including accuracy, precision, recall, F1-score, specificity, and area under the curve–receiver operating characteristic (AUC-ROC). By identifying the most effective model, this research seeks to provide healthcare professionals with a reliable tool for early DVT detection, thereby improving clinical decision-making and patient outcomes.

## 2. Materials and Methods

### 2.1. Related Work

While the use of machine learning to predict DVT is still evolving, numerous important studies have highlighted the effectiveness of these techniques in accurately diagnosing DVT and similar conditions. This section reviews key research in the field, emphasizing the machine learning methods employed and the outcomes achieved. By analyzing these studies, we gain valuable insights into the current landscape of DVT prediction, which informs the approach and aims of our own investigation into enhancing DVT detection through machine learning models.

Logan Ryan et al. [12], in their study, applied machine learning algorithms, specifically XGB, to predict DVT among hospitalized patients. The dataset, collected from over 99,000 patient records at a large academic hospital, included key clinical features, such as cancer history, venous thrombo-embolism (VTE) history, and international normalized ratio (INR). The models were designed to predict DVT onset within 12 and 24 h before occurrence. Among the tested models, XGB achieved the best performance, with an AUC-ROC of 0.85 for the 24 h prediction.

Contreras-Luján, E.E. et al. [13], in their study, applied machine learning techniques, including DT, KNN, SVM, and RF, to predict the early diagnosis of DVT. The dataset was composed of 59 real cases from a public hospital, further augmented with 10,000 synthetic examples to enhance model training. Among the models, kNN achieved the highest performance, with an accuracy of 90.4%, a precision (specificity) of 80.66%, a recall (sensitivity) of 79.78%, an F1-score of 0.80, and an AUC-ROC of 0.86.

Guan C. et al. [14], in their study, employed machine learning techniques such as RF, XGB, and SVM to predict VTE in critically ill patients. The dataset was derived from the eICU Collaborative Research Database, encompassing over 109,000 patients from 207 centers. After hyperparameter tuning and model evaluation, RF was identified as the best-performing model, achieving an AUC-ROC of 0.9378, an accuracy of 99.58%, a precision of 90.95%, a recall of 77.91%, and an F1-score of 0.8393.

Conghui Wei et al. [15], applied machine learning models to predict DVT in patients with lower extremity fractures. The models used include XGB, LR, RF, MLP, and SVM. The dataset comprised patients from the Second Affiliated Hospital of Nanchang University, with 4424 cases, including 207 DVT cases. The XGB model achieved the best performance with an AUC-ROC of 0.979, an accuracy of 93.1%, a sensitivity of 95.6%, and a specificity of 91.1%.

Hou et al. [16], in their study, employed machine learning techniques such as SVM, LR, DT, RF, and the ANN to predict the risk of DVT in rehabilitation inpatients. The dataset consisted of 801 patients, 71 of whom were diagnosed with DVT. Of all the models tested, the ANN provided the best performance, achieving an AUC-ROC of 0.97, making it the most stable and accurate method for predicting DVT. Key metrics for the ANN included an accuracy of 95%, sensitivity of 92%, specificity of 96%, precision of 93%, and an F1-score of 0.92.

In the study by Namjoo-Moghadam et al. [17], the authors used machine learning algorithms to predict the diagnosis of CVT based on clinical data. The dataset was collected from the Iran Cerebral Venous Thrombosis Registry and included 314 CVT cases and 575 control patients. The following four machine learning models were evaluated: GLM, RF, SVM, and XGB. Among these, the SVM model achieved the best performance with an AUC-ROC of 0.910, a recall of 0.73, and a precision of 0.88.

Our study conducted a systematic evaluation of various machine learning models to predict the risk of DVT in hospitalized patients. We utilized a diverse set of models, including LR, RF, XGB, ANN, kNN, GB, CatBoost, and LightGBM, to provide a deeper understanding of DVT risk assessment beyond conventional methods. Through meticulous hyperparameter tuning and cross-validation, we ensured that each model was optimized for maximum predictive accuracy. This comprehensive approach enabled a detailed assessment of each model’s performance in predicting DVT risk.

In the methodology section, we examine the strengths and weaknesses of the machine learning models used, providing a thorough analysis of their predictive capabilities. The following section offers a comparative assessment of the models’ performance, evaluated using key metrics such as accuracy, precision, recall, F1-Score, specificity, and AUC-ROC. By integrating these results, we aim to deepen the understanding of machine learning’s potential in predicting DVT, while equipping clinicians with tools to enhance patient care through early detection and timely intervention.

Taking into account predictive factors, patient behavior and comorbidities can help us avoid long-term stress. By implementing early intervention and providing continuous care, we can improve the quality of life [18,19,20].

Our findings underscore the potential of machine learning models in identifying DVT risk, highlighting key aspects of their practical application in clinical settings.

### 2.2. Proposed Methodology

The purpose of this study is to develop predictive models for assessing the risk of DVT using a comprehensive set of eight machine learning algorithms, as follows: LR, RF, LGBM, GB, kNN, XGB, CB, and ANN. These models will be trained and validated using clinical and demographic data from patients, aiming to accurately predict the likelihood of thrombosis. The models’ performance will be thoroughly evaluated by comparing key metrics such as accuracy, precision, recall, F1-score, specificity, and ROC-AUC. By analyzing these metrics, the study seeks to identify the most effective machine learning model for predicting DVT, which could aid in early diagnosis and enhance clinical decision making. The comparative evaluation of these algorithms will provide insights into their applicability and potential for integration into clinical practice for the prevention and management of DVT.

#### 2.2.1. Data Collection

The dataset for this study, comprising 299 records with 69 variables, was collected from the ‘Saint Apostle Andrew’ County Emergency Clinical Hospital, Galati. Initially provided in Excel format, it contained a range of demographic, clinical, and laboratory data relevant to predicting DVT. The dataset includes patient-level records with key demographic variables such as age, gender (female or male), and environment (urban or rural). Clinical information includes fracture type (e.g., pelvis, femur, calf, and foot), fracture location (left or right side), and hospitalization metrics such as the number of days hospitalized and time intervals between clinical events, including admission, surgery, and embolism. Additionally, laboratory test results, including glucose, urea, creatinine, and bilirubin levels, as well as detailed blood analysis values like RBC, HGB, and WBC, provide a comprehensive overview of the patients’ clinical profiles. The dataset was transformed into a CSV file to facilitate analysis using machine learning algorithms.

#### 2.2.2. Preprocessing

The initial dataset inspection revealed issues with non-numeric categorical columns like gender, environment, fracture type, and fracture location, which were manually encoded to numerical values for machine learning compatibility. Missing data in key clinical parameters, such as days between admission and surgery, were either imputed or removed, and formatting inconsistencies were standardized. Columns with excessive missing data, including surgery-related metrics (blood pressure, heart rate, and respiratory rate during surgery), were also removed.

The dataset includes both numerical features (e.g., age, weight, body mass index, vital signs at admission, biochemical and hematological parameters) and categorical features (e.g., gender, environment, and type and location of fracture), for a total of 49 variables, with thrombosis as the binary target variable.

After cleaning, the following categorical variables were encoded: gender as 0 (female) and 1 (male), environment as 0 (urban) and 1 (rural), fracture type as 0 (pelvis), 1 (femur), 2 (calf), and 3 (foot), and fracture location as 0 (left) and 1 (right). Additional columns with irrelevant clinical parameters like neutrophils_2 and thrombosis were removed. The final dataset was fully numeric, free of missing values, and prepared for machine learning analysis. The final dataset, now consisting of 299 rows and 49 columns, is fully numeric, free of missing values, and prepared for machine learning analysis.

To address the class imbalance in the dataset, we implemented the SMOTEEN technique, a combination of SMOTE and ENN, which is effective for both oversampling the minority class and cleaning noisy data. In the original dataset, the class distribution was highly imbalanced, with 41 instances of class 1 (thrombosis cases) and 258 instances of class 0 (non-thrombosis cases), making it difficult for machine learning models to accurately predict the minority class.

Using SMOTE, we synthetically generated new instances for the minority class (class 1), increasing its representation in the dataset. Subsequently, ENN was applied to remove noisy and borderline examples from the majority class (class 0). This process resulted in a new dataset with a more balanced distribution, containing 229 instances of class 1 and 149 instances of class 0, for a total of 378 rows.

This transformation helped ensure that machine learning models had a more balanced representation of both classes during training, improving their ability to generalize to the minority class. While SMOTEEN addresses class imbalance by generating synthetic samples, caution must be exercised to avoid potential overfitting, as the synthetic data are derived from existing patterns, which could lead to redundancy.

**Figure 1:**
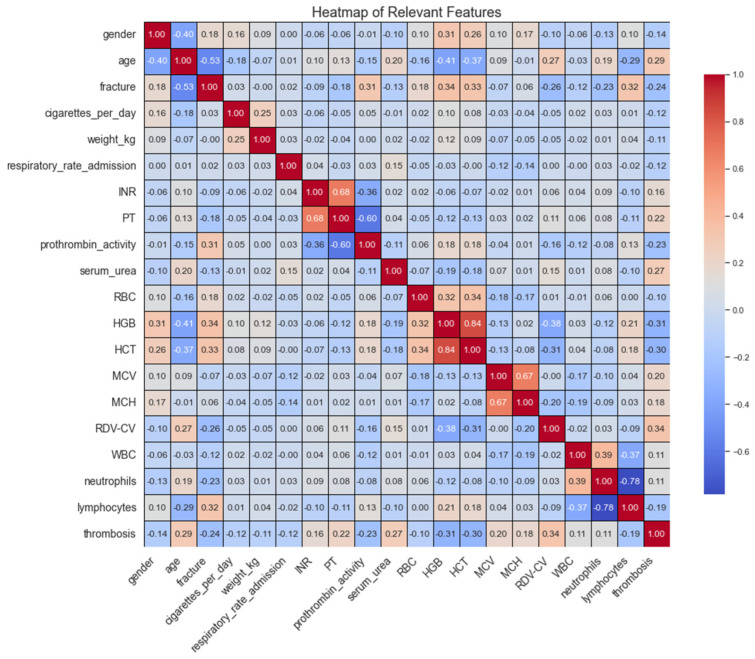
Correlation heatmap showing correlations among clinical features, highlighting relationships that may influence DVT predictions.

A heatmap was used to visualize the relationships among key features in the dataset, revealing several noteworthy correlations. Age is negatively associated with HGB and HCT, indicating that older patients tend to have lower levels of these blood markers. Additionally, fracture type shows a moderate correlation with thrombosis, suggesting that certain types of fractures may elevate the risk of thrombosis. Clotting factors such as INR and PT also display a positive correlation with thrombosis, reflecting their roles in blood coagulation. Another significant relationship is observed between MCV and RDV-CV, highlighting how variations in red blood cell size are linked to increased cell volume. These correlations underscore the relevance of these clinical and demographic features in predicting thrombosis and guide their inclusion in the machine learning models shown in Figure 1.

After completing the preprocessing steps and exploratory data analysis, the dataset was split into training and testing subsets in a 70:30 ratio, a commonly used standard in machine learning. This split allocated 70% of the data for training, enabling the model to learn underlying patterns, while the remaining 30% was reserved for testing to assess its performance on unseen data. This approach is designed to ensure the model generalizes effectively to new data, reducing the likelihood of overfitting or underfitting—two frequent challenges in predictive modeling.

The objective of our study is to predict DVT by analyzing the relationships between various clinical and demographic factors and the target outcome, thrombosis. To achieve this, we implemented the following eight supervised machine learning models: LR, RF, LGBM, GB, kNN, XGB, CB, and ANN. The performance of these models was influenced by key factors, such as data quality, feature selection, hyperparameter tuning, and the balance between overfitting and underfitting. Ensuring the interpretability of the models was also essential for generating reliable predictions and improving their practical utility in clinical settings.

We used Grid Search CV to optimize the hyperparameters for each algorithm. This method systematically evaluates different combinations of hyperparameters through cross-validation, minimizing the risk of overfitting to specific data subsets. Once the optimal hyperparameters were identified, the models were retrained on the full training dataset and tested on a separate test set to evaluate their performance on new, unseen data that were not used during training, thereby assessing their ability to make accurate predictions on future or unknown cases.

For LR, GridSearchCV explored a range of hyperparameters, including regularization strength (C), penalties (l1, l2, and elasticnet), solvers, and the number of maximum iterations (max_iter). The best parameters—C = 1, penalty = ‘l1’, solver = ‘liblinear’, and max_iter = 100—were selected based on the cross-validation. With these settings, the final LR model showed improved performance in accuracy, precision, recall, F1-score, specificity, and ROC-AUC.

For the RF model, GridSearchCV explored a range of hyperparameters, including the number of trees (n_estimators), maximum tree depth (max_depth), minimum samples required to split a node (min_samples_split), minimum samples required at a leaf node (min_samples_leaf), and whether bootstrap sampling was used (bootstrap). The best parameters—n_estimators = 100, max_depth = 30, min_samples_split = 2, min_samples_leaf = 1, and bootstrap = True—were selected based on cross-validation. Using these settings, the final random forest model demonstrated improved performance across key metrics, including accuracy, precision, recall, F1-score, specificity, and ROC-AUC.

For the LGBM model, GridSearchCV was used to explore a range of hyperparameters, including the number of boosting iterations (n_estimators), learning rate (learning_rate), maximum tree depth (max_depth), number of leaves (num_leaves), and minimum samples per leaf (min_child_samples). Based on cross-validation, the best parameters identified were boosting_type = ‘gbdt’, learning_rate = 0.1, max_depth = 10, min_child_samples = 30, n_estimators = 200, and num_leaves = 31. With these optimal settings, the final LGBM model demonstrated improved performance across key metrics, including accuracy, precision, recall, F1-score, specificity, and ROC-AUC, ensuring its effectiveness in predicting deep venous thrombosis (DVT).

For the GB model, GridSearchCV explored a range of hyperparameters, including the number of boosting stages (n_estimators), learning rate (learning_rate), maximum tree depth (max_depth), minimum samples per leaf (min_samples_leaf), minimum samples to split a node (min_samples_split), and the fraction of samples used for training each base learner (subsample). The best parameters identified were learning_rate = 0.1, max_depth = 3, min_samples_leaf = 4, min_samples_split = 2, n_estimators = 300, and subsample = 1.0. With these optimized settings, the final GB model showed improved performance across key metrics, including accuracy, precision, recall, F1-score, specificity, and ROC-AUC, making it a reliable model for predicting DVT.

For the k-NN model, GridSearchCV was used to explore various hyperparameters, including the number of neighbors (n_neighbors), the distance metric (metric), and the weighting scheme (weights). Based on cross-validation, the best parameters were identified as n_neighbors = 3, metric = ‘manhattan’, and weights = ‘uniform’. Using these optimal settings, the final k-NN model demonstrated improved performance across key metrics, including accuracy, precision, recall, F1-score, specificity, and ROC-AUC, confirming its utility in predicting DVT.

For the XGB model, GridSearchCV was employed to fine-tune a range of hyperparameters, including the number of boosting rounds (n_estimators), learning rate (learning_rate), maximum tree depth (max_depth), minimum child weight (min_child_weight), subsample ratio (subsample), and column sampling ratio (colsample_bytree). After cross-validation, the optimal parameters were identified as n_estimators = 100, learning_rate = 0.1, max_depth = 3, min_child_weight = 1, subsample = 0.8, and colsample_bytree = 0.8. These settings were used to train the final XGB model, which achieved improved performance across multiple evaluation metrics, including accuracy, precision, recall, F1-score, specificity, and ROC-AUC. This robust tuning and cross-validation approach ensured that the model was both accurate and generalizable in predicting deep venous thrombosis (DVT).

For the CB model, GridSearchCV was applied to tune several hyperparameters, including the number of iterations (iterations), learning rate (learning_rate), tree depth (depth), L2 regularization (l2_leaf_reg), number of splits for categorical features (border_count), and subsample ratio (subsample). The optimal hyperparameters identified were iterations = 100, learning_rate = 0.2, depth = 4, l2_leaf_reg = 1, border_count = 32, and subsample = 1.0. These settings were used to train the final CB model, which demonstrated improved performance across key metrics, including accuracy, precision, recall, F1-score, specificity, and ROC-AUC, ensuring the model’s effectiveness in predicting DVT.

For the ANN, the model we utilized is the Keras Sequential model, specifically designed for binary classification tasks. This architecture is configured to balance performance and prevent overfitting by leveraging a combination of dense layers, dropout, and batch normalization.

The first layer is a dense layer with 128 units, activated using the ReLU activation function, followed by a dropout layer with a rate of 0.3, which helps in reducing overfitting by randomly deactivating 30% of neurons during training. To stabilize and accelerate training, batch normalization is applied, which normalizes the activations at each layer.

Following this, the architecture includes a second dense layer with 32 units, again activated by the ReLU function, followed by another dropout layer with a 0.3 rate and batch normalization. This layered structure allows the model to learn increasingly abstract and complex features from the input data.

The final layer is a single-unit dense layer with a sigmoid activation function, designed to produce a binary output for classification. The model is compiled with the Adam optimizer, which adapts the learning rate dynamically during training, and the MSE loss function, which provided better results for this dataset compared to binary cross-entropy.

To prevent overfitting, Early Stopping is employed, which halts training if the validation loss does not improve for a set number of epochs, ensuring the model does not overtrain on the data. The combination of these elements allows the ANN to achieve highly accurate classifications while minimizing overfitting, leading to strong generalization on the validation set.

#### 2.2.3. Experimental Setup

To conduct the simulations, we leveraged a robust computational infrastructure consisting of eight VMs hosted on a server powered by AMD EPYC 7713 processors (Advanced Micro Devices, Inc. (AMD), Santa Clara, CA, USA) operating at a frequency of 3.675 GHz. Each VM was specifically optimized for high-performance computing tasks, equipped with 6 cores and 512 GB of RAM, providing the necessary computational power to process large datasets and execute complex machine learning models without performance bottlenecks. In addition, each virtual machine was equipped with a 100 GB HDD, offering ample storage capacity for handling simulation data, intermediate results, and final outputs. This setup ensured that we could run multiple simulations concurrently, allowing for comprehensive hyperparameter tuning and model evaluation in parallel. The reliability of this infrastructure was key to producing consistent and reproducible results across all experiments, enabling efficient and seamless execution of the entire simulation workflow.

### 2.3. System Architecture

The system architecture used in this study followed a clear workflow, starting from data acquisition and feature selection, followed by preprocessing to clean and structure the data for analysis. The dataset was then split into training and validation sets (70:30), and machine learning algorithms, such as LR, RF, LGBM, GB, kNN, XGB, CB, and an ANN, were trained and evaluated on this setup.

Hyperparameter tuning plays an important role in this architecture, fine-tuning each algorithm to optimize performance. Once trained, the models are evaluated to predict the target outcomes efficiently and accurately.

The system architecture for model development and testing was implemented within the Spyder IDE, using Python as the primary programming language. Key libraries, such as Pandas for data manipulation, NumPy for numerical processing, TensorFlow for deep learning, and Scikit-Learn for machine learning algorithms, were integrated into the workflow to streamline data processing, model training, and evaluation.

#### 2.3.1. Evaluation Metrics

To evaluate the performances of the predictive models developed in this study, the following six commonly utilized evaluation metrics were employed: precision, recall, specificity, accuracy, F1-score, and the ROC-AUC. Each of these metrics plays a critical role in assessing the effectiveness of binary classifiers.

Precision measures the proportion of correctly identified positive cases out of all cases predicted as positive, making it a valuable metric in scenarios where false positives are costly. Recall, on the other hand, evaluates the model’s ability to correctly identify all actual positive cases, ensuring that the model does not miss critical positive predictions. Specificity is equally important as it measures the ability to correctly identify negative cases, reducing the occurrence of false positives.

Accuracy provides a broader view, reflecting the overall correctness of the model by calculating the proportion of true results, both positive and negative, across the entire dataset. The F1-score balances precision and recall, offering a harmonic mean of the two, which is particularly useful when the class distribution is imbalanced. Finally, the ROC-AUC score provides insight into the model’s ability to distinguish between positive and negative classes across various threshold settings, offering a comprehensive measure of classification performance.

These evaluation measures are not only standard in predictive modeling but are also widely applied in domains such as disease diagnosis, fraud detection, and spam filtering, making them well-suited for assessing the accuracy and reliability of the models in this study. By using a combination of these metrics, a more holistic and robust evaluation of model performance is ensured.

Definitions:

The following terms are used to describe the performance of machine learning models:

- *TP* (True Positives): The number of positive instances correctly predicted by the model;
- *FP* (False Positives): The number of negative instances incorrectly predicted as positive by the model;
- *TN* (True Negatives): The number of negative instances correctly predicted by the model;
- *FN* (False Negatives): The number of positive instances incorrectly predicted as negative by the model.

*Precision*:

(1) $$Precision=\frac{TP}{TP+FP}$$

*Precision* measures the proportion of true positives (*TP*) among all predicted positives (*TP* + *FP*), indicating how accurate the positive predictions are.

*Recall* (*Sensitivity*):

(2) $$Recall=\frac{TP}{TP+FN}$$

*Recall* measures the proportion of actual positives correctly identified (*TP*) out of all true positives (*TP* + *FN*), reflecting the model’s ability to detect positive cases.

*Specificity* (True Negative Rate):

(3) $$Specificity=\frac{TN}{TN+FP}$$

*Specificity* measures the proportion of actual negatives correctly identified (*TN*) out of all true negatives (*TN* + *FP*), showing the model’s effectiveness in identifying negative cases.

*Accuracy*:

(4) $$Accuracy=\frac{TP+TN}{TP+TN+FP+FN}$$

*Accuracy* reflects the proportion of correctly predicted instances (both true positives and true negatives) out of all predictions.

*F1*-Score:

(5) $$F1\text{-}score=2\;\times \;\frac{Precision\;\times \;Recall}{Precision+Recall}$$

*F1*-Score is the harmonic mean of precision and recall, balancing the trade-off between false positives and false negatives, especially useful when classes are imbalanced.

*ROC*-*AUC* (Area Under the Receiver Operating Characteristic Curve):

(6) $$ROC\text{-}AUC=\int_{0}^{1}TPR(FPR)d(FPR)$$

where

- *TPR* = $\frac{TP}{TP+FN}$ is the true positive rate;

- *FPR* = $\frac{FP}{FP+TN}$ is the false positive rate.

The *ROC*-*AUC* score measures the area under the *ROC* curve, representing the trade-off between the true positive rate (*TPR*) and false positive rate (*FPR*) across various thresholds. It is a comprehensive metric of model performance.

#### 2.3.2. Software

For the development and optimization of our machine learning models, we utilized Spyder IDE version 5.5.1 in combination with Python version 3.11.9 as the primary platform. Spyder provided a powerful yet user-friendly environment, which greatly facilitated the process of writing, debugging, and executing Python scripts. Its intuitive interface and integrated development tools allowed for seamless handling of complex machine learning workflows.

Leveraging Spyder’s robust features, we implemented various machine learning algorithms, ranging from traditional models like logistic regression and random forest to more advanced methods such as Gradient Boosting and artificial neural networks. The IDE’s integrated libraries, such as Pandas, NumPy, TensorFlow, and Scikit-Learn, enabled us to efficiently manage data preprocessing, feature engineering, and model training tasks.

Additionally, Spyder’s support for tools like GridSearchCV was invaluable in performing hyperparameter tuning, allowing us to systematically search for the optimal parameters that maximized model performance. This functionality, combined with real-time code execution and debugging features, ensured that we could iteratively refine and evaluate our models, ultimately enhancing their predictive accuracy and robustness. The combination of Python’s versatility and Spyder’s advanced debugging, profiling, and variable exploration tools made it an ideal environment for machine learning model development and optimization.

## 3. Results

The machine learning models developed for predicting DVT were rigorously evaluated using a range of performance metrics, including AUC-ROC, accuracy, F1-score, precision, recall, and specificity. These metrics were chosen to provide a comprehensive assessment of each model’s predictive capabilities, ensuring a balance between identifying true positive cases of DVT and minimizing false positives. The clinical dataset, comprising patient data collected from the St. Andrew County Emergency Clinical Hospital, formed the foundation for training and testing these models.

The dataset included a variety of demographic, clinical, and laboratory features critical for predicting DVT, making it well-suited for machine learning algorithms. Prior to training, each model underwent a process of hyperparameter tuning through cross-validation, ensuring that the chosen parameters would maximize the models’ performance across all relevant metrics. This tuning process is vital for fine-tuning the models’ capacity to generalize well to unseen data, thereby preventing overfitting and underfitting issues.

Table 1 summarizes the key results of each model after hyperparameter tuning. These results reflect the models’ ability to accurately diagnose DVT based on the features provided in the dataset, highlighting the strengths and limitations of each approach. The models were tested under the same conditions to ensure a fair comparison, providing insights into their suitability for clinical application in predicting DVT.

LR exhibited the highest accuracy at 0.97, indicating its strong overall performance in classifying both positive and negative cases. Other models, including LGBM, XGBt, and ANN, maintained high accuracy at 0.96, making them reliable choices for DVT diagnosis.

The F1-score, which balances precision and recall, highlights LR’s standout performance, with an F1-score of 0.98 due to its perfect recall (0.99) and high precision (0.95). Models such as LGBM and the ANN also achieved high F1-scores, making them robust alternatives for DVT prediction.

When it comes to precision, XGB (0.95) and LR (0.95) lead, indicating their ability to make accurate predictions with few false positives. This is particularly critical in medical diagnosis, where overdiagnosis can lead to unnecessary interventions.

Recall, or the model’s sensitivity to identifying DVT cases, was perfect for logistic regression, meaning it correctly identified all positive cases, which is essential in clinical settings, where missing a DVT case could have serious consequences. Other models, including LGBM, kNN, and ANN, also demonstrated high recall, underscoring their effectiveness in DVT detection.

Specificity, which measures a model’s ability to correctly identify negative cases (non-DVT), showed that LR and XGB performed best with 0.94, making them reliable at minimizing false positives. RF and LGBM also performed well, with specificity scores of 0.92.

Across all metrics, LR consistently delivered excellent performance, particularly excelling in recall and accuracy. RFt and k-NN also provided strong results, particularly in distinguishing between positive and negative cases, with high AUC-ROC scores.

Models such as LGBM, XGB, and the ANN displayed balanced performance across precision, recall, and accuracy, making them reliable options for DVT prediction. CB and GB, while effective, exhibited slightly lower specificity, which may result in a higher number of false positives.

**Figure 2:**
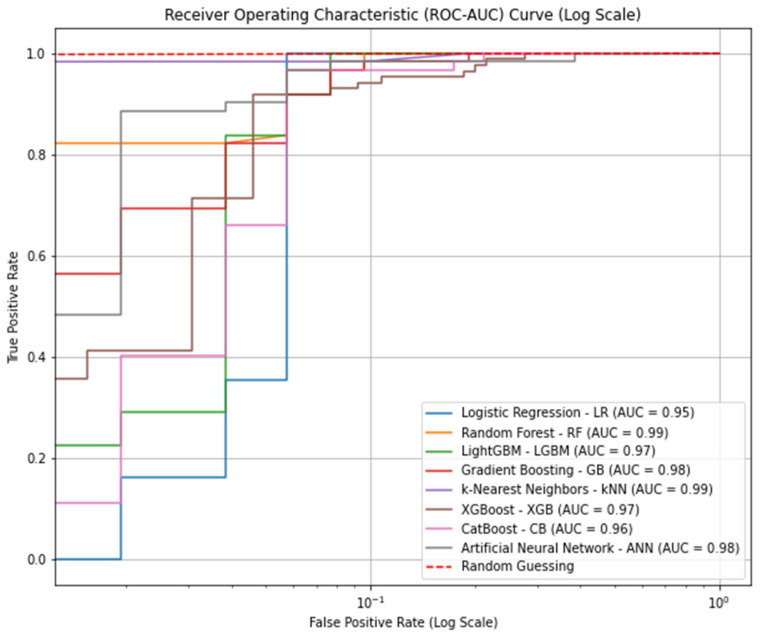
Receiver operating characteristic (ROC) curve. The ROC curve compares the performance of eight machine learning models in predicting DVT, with AUC values indicating their accuracy.

The ROC-AUC curve displayed in Figure 2 provides a comprehensive comparison of the performance of eight machine learning models in predicting DVT. Each model’s curve illustrates the trade-off between the true positive rate (sensitivity) and the false positive rate, while the AUC values reflect the overall ability of the models to distinguish between DVT-positive and -negative cases. Among the models, kNN (AUC = 0.99) and RF (AUC = 0.99) showed the highest discriminative power, closely followed by the ANN (AUC = 0.98). The other models, including GB (AUC = 0.98), XGB (AUC = 0.97), and LGBM (AUC = 0.97), also exhibited strong performances. LR shows a slightly lower, yet still robust AUC of 0.95. The ROC curve is plotted using a log–log scale, which enhances the visualization of a model’s performance, particularly at low false positive rates. This visualization clearly demonstrates that all models performed well, with most achieving high AUC scores, signifying their effectiveness in predicting DVT with minimal false positives.

**Figure 3:**
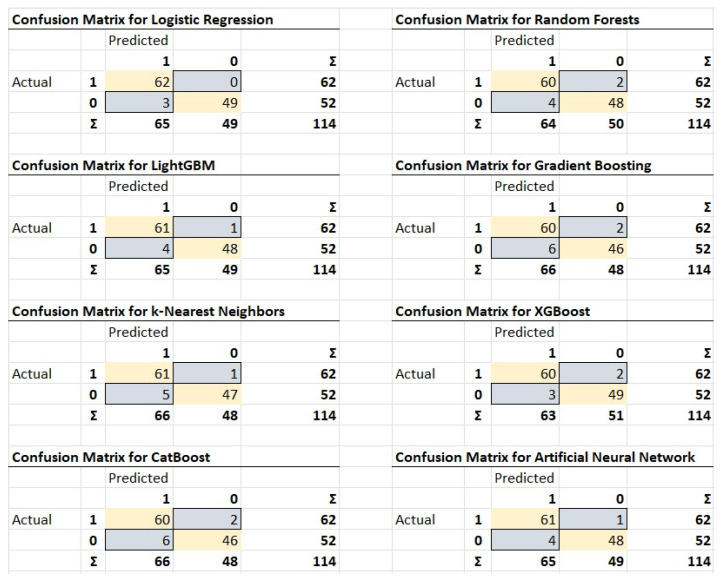
Confusion matrices showing the true/false positives and negatives for each model, providing insights into prediction accuracy and errors.

Figure 3 presents the confusion matrices for the eight machine learning models developed to predict DVT. Each matrix offers a detailed comparison of the models’ predicted outcomes against the actual results, showcasing their effectiveness in correctly classifying both positive and negative DVT cases.

For all models, the top-left cell represents the TP (patients with DVT correctly identified), while the bottom-right cell represents TN (patients without DVT correctly identified). The top-right cell shows FP (patients without DVT incorrectly predicted as having DVT), and the bottom-left cell shows FN (patients with DVT incorrectly predicted as not having DVT).

Across the models, LR achieved perfect precision and recall for DVT cases (62 TP, 0 FN), with just three FP. RF, LGBM, and XGB similarly demonstrated high performance, with only slight differences in the number of FP and FN cases. Models such as the kNN and CBoost performed slightly less well, with higher FP and FN counts, particularly in the non-DVT class.

The confusion matrices help to understand the specific misclassifications made by each model, providing insight into how well the models balance sensitivity (recall) and specificity. Most models show strong performance, particularly in identifying DVT cases, with relatively few false negatives, indicating their potential clinical usefulness in predicting DVT.

The results for the various machine learning models demonstrate their strong potential in predicting DVT with high accuracy and reliability. Each model was carefully evaluated using a range of metrics, including AUC-ROC, accuracy, precision, recall, F1-score, and specificity, ensuring a comprehensive assessment of their performance. While models like kNN, RF, and the ANN showed slightly higher AUC-ROC scores, all models displayed excellent balance between correctly identifying true positives and minimizing false negatives. The confusion matrices provide further insights into the models’ classification abilities, illustrating their potential utility in a clinical setting for early and accurate DVT detection. These results underscore the importance of data-driven approaches in medical diagnostics, offering a foundation for future improvements and real-world applications of machine learning in healthcare.

## 4. Discussion

The results of this study demonstrate the potential of machine learning algorithms in predicting DVT based on clinical data. Among the models, kNN, RF, and the ANN performed notably well, achieving high AUC-ROC scores, which indicate a strong capacity to differentiate between patients with and without DVT. While these models showed superior overall performance, others, such as LR and CB, exhibited good but slightly lower predictive accuracy, especially in terms of sensitivity—an important metric in clinical settings where false negatives could result in undetected DVT, with potentially serious complications.

The kNN and RF models achieved the highest AUC-ROC values, underscoring their ability to balance sensitivity and specificity. Both models performed exceptionally well in identifying true positive cases, making them valuable for early DVT detection. However, these models were associated with higher false positive rates, which could lead to unnecessary follow-up tests. Although this may burden healthcare resources, the benefit of higher sensitivity, ensuring fewer missed DVT cases, outweighs the risk of false positives in critical care environments.

The ANN also delivered strong results, particularly in recall and precision metrics. Its ability to capture complex, non-linear patterns in the data, which simpler models like LR may overlook, might explain its success in this context. However, the complexity of ANN models requires caution, especially when considering overfitting and computational demands. Given these challenges, the ANN model’s robustness suggests it is well-suited for handling the inherent variability of clinical data, though additional validation is necessary to ensure reliability in broader clinical use.

Despite its simplicity, the LR model demonstrated good predictive performance, though it did not reach the accuracy of more complex models like RF and ANN. However, its interpretability and ease of use make it a valuable option in scenarios where model transparency is a priority, such as in real-time decision support or when computational resources are limited. The LR model could be a practical choice for baseline applications in clinical environments where simplicity and speed are critical.

The practical implications of these findings are significant for clinical workflows. The high sensitivity rates observed in models like kNN, RF, and the ANN suggest that these algorithms could enhance early DVT detection, potentially reducing the incidence of severe complications such as pulmonary embolism. Early detection through these models would be particularly useful in both emergency care and routine screening in high-risk populations.

The use of confusion matrices also provided important insights into the strengths and weaknesses of each model. This analysis highlighted the trade-offs between false positives and false negatives. While false positives can lead to additional but unnecessary testing, false negatives pose a greater risk, as missing a DVT diagnosis can have critical consequences. Models like RF and LR demonstrated a good balance in minimizing both types of errors, which reinforces their potential utility in clinical practice.

However, this study has several limitations. The relatively small size of the dataset (299 records) may limit the generalizability of the findings. A larger, more diverse dataset, sourced from multiple hospitals, would provide a better representation of the general population and likely improve model robustness. Additionally, the dataset was derived from a single center, which may introduce selection bias and limit the external validity of the models.

Furthermore, while we optimized the models using Grid Search CV, further improvements in hyperparameter tuning are possible. Techniques such as Bayesian optimization or random search could potentially yield better model performance by exploring a broader range of hyperparameters. Incorporating domain-specific knowledge, such as risk factors for DVT, into the feature selection process could also improve the models’ interpretability and predictive accuracy.

Finally, integrating these machine learning models into clinical practice would require addressing several practical challenges. These include model explainability, integration with electronic health records (EHR) systems, and ensuring that clinicians are adequately trained to interpret and use model outputs. Additionally, prospective clinical trials would be essential to validate the real-world effectiveness of these models before they can be adopted widely in healthcare settings.

In summary, the findings from this study indicate that machine learning models, particularly kNN, RF, and ANN, show great promise in predicting DVT. Future research should focus on expanding the dataset, improving model optimization techniques, and testing these models in real-world clinical environments to fully realize their potential in improving patient outcomes.

Table 2 presents a summary of the research studies, highlighting the proposed models along with their respective performance metrics.

## 5. Conclusions

This study explored the application of machine learning models for the prediction of DVT using clinical data. A range of models, including LR, RF, LGBM, GB, kNN, XGB, CB, and ANN, were trained and evaluated using a clinical dataset obtained from Galati, St. Andrew County Emergency Clinical Hospital. Through extensive hyperparameter tuning and performance evaluation, using key metrics, such as AUC-ROC, accuracy, precision, recall, F1-score, and specificity, we were able to identify the strengths and weaknesses of each model in detecting DVT.

The results show that the LR model achieved the highest recall (0.99), making it the most suitable model in terms of clinical utility, as it successfully identified all cases of DVT. This is particularly important in the medical field, where minimizing false negatives is crucial to ensuring patient safety. However, the kNeN model outperformed the other models in terms of the AUC-ROC (0.99), indicating its superior ability to distinguish between true positive and false positive cases. Both models demonstrate significant potential for clinical application, with each having specific strengths depending on the priority—whether it is minimizing missed diagnoses or balancing sensitivity and specificity.

The confusion matrices further supported these findings, providing a clear breakdown of each model’s classification performance, particularly in distinguishing between true positive and false negative cases. While some models, such as RF and ANN, also showed strong performance, LR’s perfect recall makes it a more reliable choice for ensuring that all DVT cases are correctly identified.

In summary, while several models performed well in predicting DVT, LR stood out as the most clinically useful model due to its perfect ability to detect DVT cases. However, a key limitation of this study is the relatively small size of the dataset (299 records), which may affect the generalizability of the results. Future work should focus on expanding the dataset, ideally incorporating data from multiple centers, to enhance the robustness and external validity of the findings. Additionally, integrating these models into clinical workflows and validating their performance in real-world clinical settings will be essential. By doing so, machine learning has the potential to greatly enhance patient care and improve diagnostic accuracy in DVT detection.

## Figures and Tables

**Table 1:** Evaluation scores.

Model	Accuracy	Precision	Recall	F1-Score	Specificity	AUC-ROC
Logistic Regression	0.97	0.95	0.99	0.98	0.94	0.95
Random Forest	0.95	0.94	0.97	0.95	0.92	0.99
LightGBM	0.96	0.94	0.98	0.96	0.92	0.97
Gradient Boosting	0.93	0.91	0.97	0.94	0.88	0.98
k-Nearest Neighbors	0.95	0.92	0.98	0.95	0.90	0.99
XGBoost	0.96	0.95	0.97	0.96	0.94	0.97
CatBoost	0.93	0.91	0.97	0.94	0.88	0.96
Artificial Neural Network	0.96	0.94	0.98	0.96	0.92	0.98

**Table 2:** Performance of previous work.

Authors (Year)	Dataset Collection (Samples)	Applied Models	Performance (Proposed Model)
Ryan L. et al. (2021) [12]	Academic Medical Center USA (2011–2017)	XGBoost (proposed)	Recall: 99.90% Specificity: 80.00% AUC: 0.85
Contreras-Luján E.E. et al. (2022) [13]	Unspecified Public Hospital (59 patients)	Decision Trees, K-Nearest Neighbors (proposed), Support Vector Machine, Random Forest, Multilayer Perceptron Neural Network, Extra Trees	Accuracy: 90.40% Specificity: 80.66% Recall: 79.78% AUC: 0.868
Guan C., et al. (2023) [14]	eICU Collaborative Research Database (109,000 patients)	Random Forest (proposed), eXtreme Gradient Boosting (XGBoost), Support Vector Machines	Accuracy: 99.58% Precision: 90.95% Recall: 77.91% F1-Score: 0.8393 AUC: 0.9378
Wei C. et al. (2024) [15]	Second Affiliated Hospital of Nanchang University (4424 patients)	Extreme Gradient Boosting (proposed), Logistic Regression, Random Forest, Multilayer Perceptron, Support Vector Machine	Accuracy: 93.10% Recall: 95.60% Specificity: 91.10% F1-Score: 0.942 AUC: 0.979
Hou T. et al. (2023) [16]	Affiliated Hospital of Nantong University (801 patients)	Support Vector Machine, Logistic Regression, Decision Tree, Random Forest, Artificial Neural Networks (proposed)	Accuracy: 95.00% Recall: 92.00% Specificity: 93.00% F1-Score: 0.92 AUC: 0.97
Namjoo-Moghadam A. et al. (2024) [17]	Iran Cerebral Venous Thrombosis Registry (889 patients)	Generalized Linear Model, Random Forest, Support Vector Machine (proposed), Extreme Gradient Boosting	Accuracy: 86.00% Recall: 73.00% Specificity: 88.00% F1-Score: 0.8 AUC: 0.91
Our (2024)	Galati Hospital (299 patients)	Logistic Regression (proposed), Random Forest, LightGBM, Gradient Boosting, K-Nearest Neighbors, XGBoost, CatBoost, Artificial Neural Network	Accuracy: 97.37% Precision: 95.38% Recall: 99.99% Specificity: 94.23% F1-Score: 0.9764 AUC: 0.9516
